# Facial Neuralgia

**Published:** 1849-07

**Authors:** 


### FACIAL NEURALGIA.

A tobacconist of Paris presented himself a short time back at the consultation of M. Velpeau, who had suffered intensely for eleven years, with facial neuralgia. Of so excruciating a nature were his torments, that he had only been restrained from making an attempt upon his life by the thought of the ruin it would entail on his family. He bore unequivocal marks of blisters, moxas, and setons, having been applied in great numbers, and had moreover taken a prodigious quantity of Meglin's pills. Some Surgeon had attempted a cure of the complaint by dividing one of the branches of the tri-facial nerve. He had had extracted (as it has often happened,) all the teeth of the upper jaw on the affected side. After examining him, it was found impossible to devise any method that had not already been fruitlessly employed, when a young physician connected with our publication, M. Pajol, thought of asking him if he had ever been the subject of lues venerea. He then admitted that he had had a bubo, for which he had not undergone a specific treatment.

Upon this intimation, M. Velpeau, prescribed for him a treatment with the proto-ioduret of mercury. Last Wednesday this man returned full of joy and gratitude, to announce to M. Velpeau that, for more than three weeks he had had no paroxysm, and had not suffered for a single instant. Cases of syphilitic neuralgia have been observed before, and this was a remarkable example of it, which we hope will not be lost upon the notice of the profession.

It is very probable that many osteoscopic pains are nothing else than syphilitic neuralgia, particularly when it is remembered that these pains yield sometimes in a miraculous manner, to the application of several blisters to the part affected, as M. Ricord has fully established; and that, where the blister has not fully succeeded, a deep incision over the seat of pain has answered the same intention. The efficacy of repeated blisters against neuralgia is well-known, and Dr. Marchal, of Calvi, has proved, in a work lately written, that in certain cases of cranial neuralgia, a deep incision of the parts has had a most prompt and sedative effect on the pains.—*Lancette Francaise.*

